# Points from Annual Reports

**Published:** 1921-03-19

**Authors:** 


### Points from Annual Reports.

Royal Southern Hospital, Liverpool.—The debt to the bank and other creditors on December 31, 1920, was £10,245 16s. 7d., as compared with £4,488 16s. 7d. on the corresponding date in 1919.

Bradford Royal Infirmary.—The Ladies' Linen League has collected £526 19s. 6d. for the purchase of linen. Buying of fresh supplies has been postponed in the hope that there will be a fall in prices.

Hampstead General Hospital.—During the war the debt was reduced from £7,800 to £3,200, but in the year 1920 it rose again to £8,200. It is hoped that the patients' payment scheme will help to improve matters.

Oldham Royal Infirmary.—The Endowment Fund last year was increased by £13,622, but there was a deficit of £4,892 on the year's working.

West Suffolk General Hospital, Bury St. Edmunds.—"Thanks are due to Miss Harwicke for the efficient manner in which she has carried out her duties as secretary."

The Royal Devon and Exeter Hospital, Exeter.—The average cost per occupied bed was £98 13s. 9d. in 1920, as compared with £82 9s. 7d. in 1919. Extensions estimated to cost £45,000 are contemplated.

Royal Westminster Ophthalmic Hospital.—"It is undoubtedly a fact that the hospital must look more and more to the public who come to the hospital for treatment for financial support."

Seamen's Hospital Society.—In consequence of the almost prohibitive cost of building during the past two years a beginning has not yet been made with the improvements of the old buildings at Greenwich. A bed has been endowed by the Minesweepers' Fund in memory of the Deep-Sea Fishermen who served in the Minesweeping Fleet during the Great War.

St. Andrew's Hospital, Dollis Hill.—Patients are admitted only on the recommendation of their own medical adviser. £1,000 for additional land has been given by the Lloyd's and Baltic Ambulance Unit.

